# On the Determination of Magnesium Degradation Rates under Physiological Conditions

**DOI:** 10.3390/ma9080627

**Published:** 2016-07-28

**Authors:** Eshwara Phani Shubhakar Nidadavolu, Frank Feyerabend, Thomas Ebel, Regine Willumeit-Römer, Michael Dahms

**Affiliations:** 1 Division Metallic Biomaterials, Institute of Materials Research, Helmholtz-Zentrum Geesthacht, Max-Planck-Str. 1, Geesthacht 21502, Germany; eshwara.nidadavolu@hzg.de (E.P.S.N.); frank.feyerabend@hzg.de (F.F.); thomas.ebel@hzg.de (T.E.); regine.willumeit@hzg.de (R.W.-R.); 2 Materials Technology, Hochschule Flensburg, Kanzleistraße 91–93, Flensburg 24943, Germany

**Keywords:** in vitro, degradation, long-term immersion test, simulation

## Abstract

The current physiological in vitro tests of Mg degradation follow the procedure stated according to the ASTM standard. This standard, although useful in predicting the initial degradation behavior of an alloy, has its limitations in interpreting the same for longer periods of immersion in cell culture media. This is an important consequence as the alloy’s degradation is time dependent. Even if two different alloys show similar corrosion rates in a short term experiment, their degradation characteristics might differ with increased immersion times. Furthermore, studies concerning Mg corrosion extrapolate the corrosion rate from a single time point measurement to the order of a year (mm/y), which might not be appropriate because of time dependent degradation behavior. In this work, the above issues are addressed and a new methodology of performing long-term immersion tests in determining the degradation rates of Mg alloys was put forth. For this purpose, cast and extruded Mg-2Ag and powder pressed and sintered Mg-0.3Ca alloy systems were chosen. DMEM Glutamax +10% FBS (Fetal Bovine Serum) +1% Penicillin streptomycin was used as cell culture medium. The advantages of such a method in predicting the degradation rates in vivo deduced from in vitro experiments are discussed.

## 1. Introduction

Magnesium has gained renewed interest as a potential biomaterial in recent years, mainly owing to its biodegradability and mechanical properties. With a density of 1.74 g/cm^3^, which is similar to that of human cortical bone (1.75 g/cm^3^) [1], Mg would be a preferable choice over stainless steel and titanium, which show stress shielding effect with longer implant durations inside the body. In addition, Mg is also responsible for over 300 enzymatic host—tissue interactions in the body including DNA and RNA synthesis, which makes it a necessary dietary intake nutrient (recommended daily uptake: 300 mg/day) [2]. Mg alloys exhibit positive results of tunable corrosion resistance and biocompatibility by alloying additions, microstructure modifications and chemical surface alterations. The antibacterial property of Mg alloys for clinical applications is reported to be improved after alloying with rare earths and zinc [3,4]. Surface coatings help to improve the cell adhesion and viability [5,6]. However, predicting the corrosion rates of these alloys in vivo based on experiments in vitro poses the question of how close the resemblance of in vitro testing conditions is to the real environment. Few reports suggested that the in vivo Mg degradation is many orders less compared to the in vitro results. Optimization of in vitro parameters in assessing Mg degradation so as to obtain a closer resemblance to the in vivo conditions is being carried out by the research groups worldwide. Hence, there is an ongoing discussion about the predictability of in vivo degradation behavior of magnesium and its alloys by in vitro methods [7,8]. This has led to extensive research on the optimization of in vitro methods by applying physiological conditions or flow [9,10]. However, there is still no standard procedure on how to determine predictive degradation rates in vitro.

In order to quantitatively describe corrosion, the mass loss Δm of a metallic sample is normally measured according to ASTM NACE/ASTM G31-12a standard [11]. This has to be done after t=7 days of exposure time to a well-defined electrolyte. After having determined the mass loss Δm (g), the corrosion rate (CR) is calculated according to Equation (1), where A is the surface area (cm^2^), ⍴is the density of the sample (g/cm^3^) and t is the time of immersion (h) and k = 8.79 × 10^4^, a constant which expresses corrosion rate value in mm/y:

(1) $$CR=\frac{\Delta m.\;k\;}{A\cdot t\cdot \rho }$$

**Figure 1:**
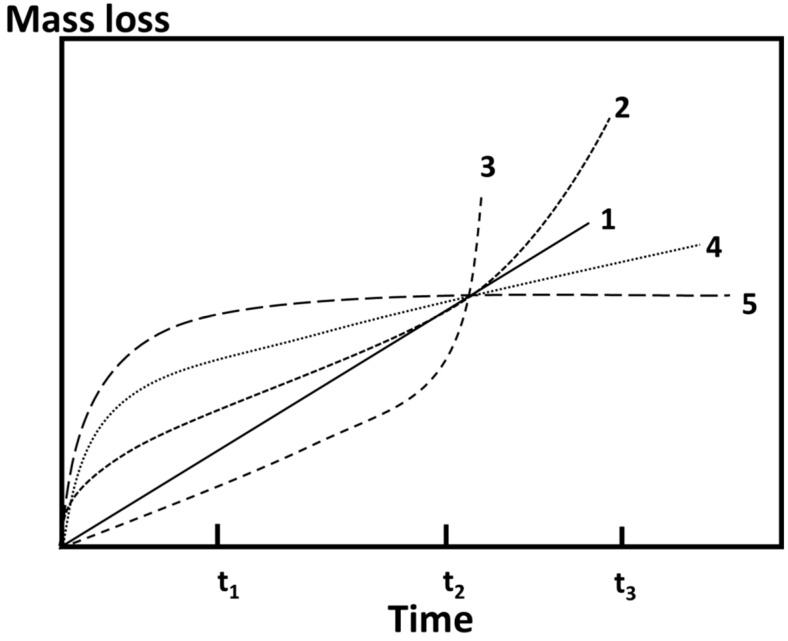
Possible mass loss vs. time curves during physiological in vitro corrosion experiments: (**1**) linear corrosion; (**2**) decreasing corrosion rate at the beginning, increasing rate at the end; (**3**) increasing corrosion rate; (**4**) decreasing corrosion rate at the beginning, linear behavior at the end; and (**5**) decreasing corrosion rate at the beginning, zero corrosion rate at the end.

The description of a process by a single parameter is useful and only useful, if the process can be modeled and mathematically described by a single parameter. This is, e.g., the case for linear dissolution of a material or parabolic growth of a layer. Then, different materials, microstructures and corrosion conditions can reasonably be compared and discussed. On the other hand, if the development of the corrosion process is unknown or differs for different materials or if the process must be mathematically described by at least a two-parameter model, and then the description of corrosion by CR may be misleading as shown in Figure 1 for different potential mass loss vs. time behaviors during corrosion experiments. At time t_1_, curve 5 would represent the quickest corrosion, followed by 4, 2, 1 and 3, respectively. At time t_2_, all measurements would exhibit the same corrosion rate, and at time t_3_, curve 3 would represent the quickest corrosion, followed by 2, 1, 4 and 5, respectively.

In order to quantitatively describe the biologically induced degradation of magnesium, the above-mentioned approach of varying immersion times has been used in numerous investigations [7,8,12,13,14,15]. Instead of standardized experiments with a fixed duration of immersion, varying exposure times to cell culture medium have been applied ranging from hours to months. Increasing exposure times within one experimental setup always showed a decreasing CR with increasing time [16,17]. So, the possible degradation curves according to Figure 1 are 2, 4 and 5. However, in the literature, it was never discussed which type of degradation curve was really applied to the corresponding in vitro test. However, with respect to long-time prediction, for medical implants on the order of a year, determining the type of degradation curve is essential. Furthermore, the degradation rates in bio-corrosion experiments are given in mm/a, implying a predictability of degradation in the order of years, which is, in fact, not true. Hence, these experiments suggest that CR is not a suitable parameter in order to properly describe the bio-degradation of magnesium. Hereof, the authors suggest defining instead of “corrosion rate” a novel term “degradation rate” DR, as derived in the following section. Please note that the term ‘corrosion rate’ is used in this paper when referring to the results demonstrated previously using ASTM NACE/ASTM G31-12a standard, and ‘degradation rate’ is referred to the current results obtained using the method described in Figure 3.

How can physiological degradation of magnesium be properly described?

Using Equation (1) and avoiding the constant:

(2) $$h=t\cdot CR=\frac{\Delta m}{A\cdot \rho }$$

The variable *h* is the mean degradation depth of the sample, a reasonable value, if the degradation process is nearly uniform. Since a rate in science is usually a derivative with respect to time, the degradation rate DR is then:

(3) $$DR=\frac{dh}{dt}=CR+t\cdot \frac{dCR}{dt}$$

The degradation rate may be approximated by numerical differentiation:

(4) $$\bar{DR_{12}}=\frac{\Delta h}{\Delta t}=\frac{1}{A\cdot \rho }\cdot \frac{\Delta m_{2}-\Delta m_{1}}{t_{2}-t_{1}}$$

The value $\bar{DR_{12}}$ is the mean degradation rate for a certain time interval. From Equation (4) the corrosion rate CR = f(t) is in fact the mean degradation rate $\bar{DR_{12}}$ for the time interval 0:t. The problem with numerical differentiation is that when using differences of small numbers, the absolute errors add up. In Equation (4), the mass changes are measured with the uncertainty of the scale. Small changes of mass thus become extremely uncertain.

## 2. Materials and Methods

### 2.1. Materials

Starting materials used for production of specimens are listed in Table 1. The spherical powders are produced by gas atomization technique.

### 2.2. Specimen Preparation and Sterilization

#### 2.2.1. Mg-2Ag Alloy

In order to obtain a fine and homogeneous grain size, ingots were cast in a permanent mold casting at 680 °C in neutral atmosphere (Ar + SF_6_) and heat treated for 6h at 420–430 °C. Then, Mg-2Ag samples were extruded at 370 °C from 30 mm diameter to 12 mm with a speed of 2.5 mm/s and a stamp advance rate of 108 (Strangpreßzentrum, Berlin, Germany). Finally, the rods were machined into discs with 10 mm diameter and 1.5 mm thickness with a mass of approximately 180 mg each disc. The samples were gamma-sterilized before immersion test procedure.

#### 2.2.2. Mg-0.3Ca Alloy

Pure Mg powder and MAP were blended in proportions relative to their weights (96.93 wt % pure Mg+ 3.07 wt % MAP) to obtain the final Mg-0.3Ca alloy powder. This powder was, in turn, stirred with cyclohexane for 5 min to obtain a homogenous particle distribution in the mix. Drying was carried out under vacuum at 200 mbar for 24 h. Cylindrical immersion test specimens of 10 mm diameter and 22 mm length were produced by applying a uniaxial surface pressure of 175 MPa using an air hydraulic (Enerpac RC55, Milwaukee, WI, USA). The entire process of blending the powders, stirring with cyclohexane and powder pressing was carried out in a glove box chamber with argon 4.6 protective atmosphere (Unilab, MBraun, Garching, Germany) to limit oxygen entrapment. Sintering of the produced green parts was carried out at 642 °C for 63 h under Ar 6.0 environment at ambient atmospheric pressure conditions (XRetort, Xerion, Germany). The crucible setup for sintering of Mg alloy was adopted from the work of Wolff et al. [18].

**Figure 2:**
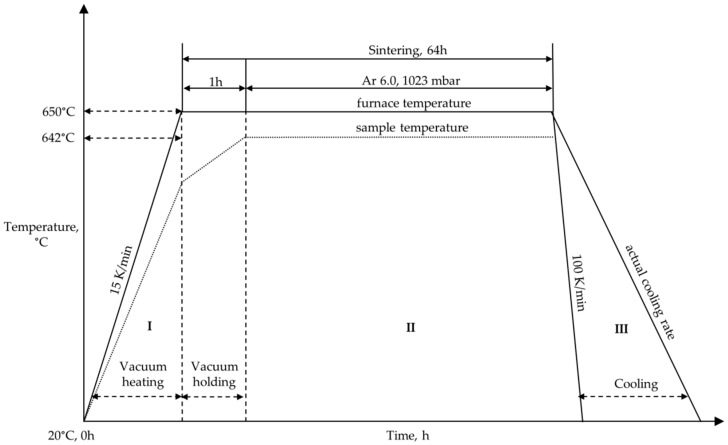
Schematic outline of sintering parameters employed during sintering run for Mg-0.3Ca alloy specimens indicating stage **I**—vacuum heating, stage **II**—sintering and stage **III**—cooling. The sample temperature is less than the set furnace temperature because of the temperature deviation in the furnace.

The sintering run consisted of three stages (Figure 2). In the first stage (I), the furnace was heated to a temperature somewhat above the sample sintering temperature of 642 °C considering the temperature deviation inside the furnace at a rate of 15 K/min under vacuum conditions. Sintering of Mg alloy took place in the second stage (II) for 1 h under vacuum in order to obtain cleaner furnace atmosphere conditions and subsequently under Ar 6.0 for 63 h. The third stage (III) involved cooling of the furnace at 100 K/min. This is, however, the set controller value and is different from the actual cooling rate of the furnace as shown in Figure 2. After sintering, the Mg-0.3Ca parts were later machined into small discs of 9 mm diameter and 2 mm thickness, which serve as immersion test specimens. Cleaning of these specimens was performed in an ultrasonic bath for 20 min using cyclohexane and acetone each as a reagent. Sterilization was followed for 5 min using 70% ethanol as reagent. Both operations were carried out in a protective hood with controlled air flow (Hohenloher Spezialmöbelwerk Schaffitzel GmbH + Co. KG, Öhringen, Germany).

### 2.3. Immersion Test

**Figure 3:**
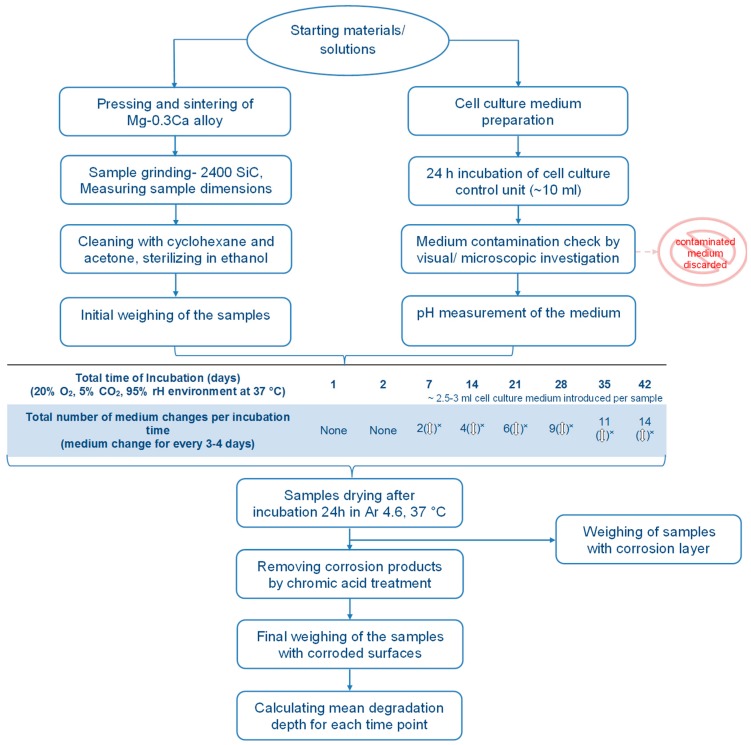
Schematic outline of immersion test procedure employed in this study for Mg-0.3Ca alloy. Sample grinding and cleaning and sterilizing processes were always followed by 24 h drying. (rH- residual humidity in the incubator; (**↕**)^x^ represents that every medium change is preceded by pH and contamination check of the fresh medium).

In order to determine the amount of degradation in the alloy, specimens were immersed in a physiological cell culture medium DMEM + Glutamax (Dulbecco’s Modified Eagle’s Medium, (+) 4.5 g/L d-Glucose, (+) Pyruvate, Life Technologies, Darmstadt, Germany) supplemented with 10% FBS (Fetal Bovine Serum, PAA Laboratories, Linz, Austria) in sterilized well-plates. In addition, a set of empty wells with cell culture medium served as control units. For degradation analysis, four specimens per incubation time were considered. Mg-2Ag samples were immersed for 3, 12 and 30 days according to the ASTM NACE/ASTM G31-12a standard. For Mg-0.3Ca, incubation times include the intervals of 1 h till 8 h followed by 1 day, 2 days, and later followed in intervals of 1 week to 6 weeks. In total, 16 mean degradation depth values were calculated for progressing incubation times for the alloy Mg-0.3Ca. Fresh cell culture medium was introduced into the well-plates for every 3–4 days to maintain constancy in pH value. Simultaneous drying process soon after incubation was carried out under argon environment at 37 °C for 24 h. The whole procedure is schematically illustrated in Figure 3.

The developed degradation product layer on the surfaces of specimens was removed with fresh chromic acid (180 g/L in distilled water, VWR International, Darmstadt, Germany). Samples were dipped in the chromic acid medium for 20 min with a gentle stir. In addition, a set of Mg-0.3Ca alloy samples in their initial state were immersed directly in chromic acid for 20 min to check the reactivity of chromic acid towards the alloy. The difference in weights observed before and after this treatment was in the range of limit of detection of the weighing machine. This confirmed the non-reactive nature of chromic acid towards the base Mg-0.3Ca alloy.

The weights of specimens recorded by using a high precision weighing scale (Sartorius, Göttingen, Germany) soon after sterilization and after chromic acid treatments were used to calculate their respective mean degradation depths using Equation (2).

## 3. Results and Discussion

**Figure 4:**
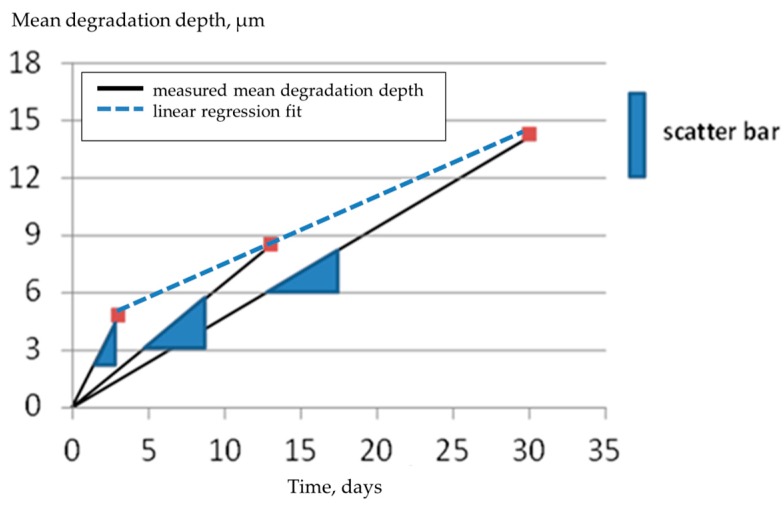
Mean degradation depth vs. time for Mg-2Ag, extruded, gamma-sterilized, exposed to DMEM Glutamax+ 10% FBS cell culture medium (**blue** triangles: CR-analysis; **blue** line: linear regression fit for measured data).

**Figure 5:**
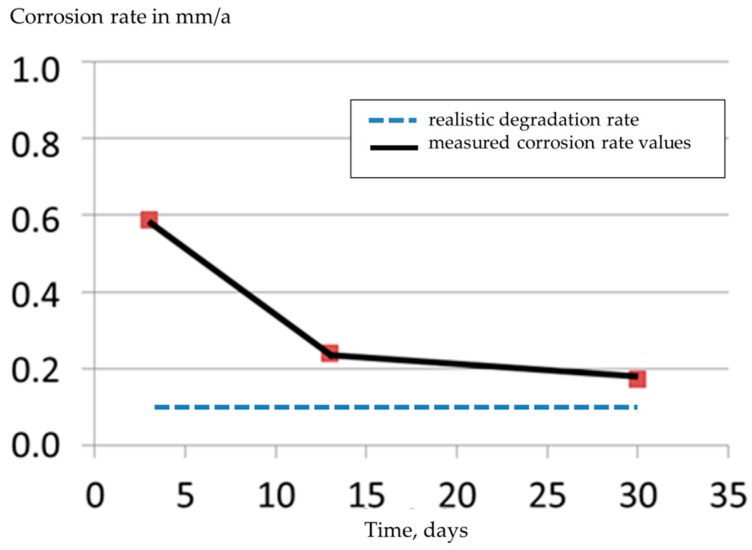
Corrosion rate vs. time for Mg-2Ag, extruded, gamma-sterilized, exposed to DMEM Glutamax+ 10% FBS cell culture medium (**blue** line: nearly constant realistic degradation rate DR = 0.1 mm/a of Mg-2Ag alloy in the time interval of 3–30 days).

To determine the mean degradation depth of Mg-2Ag alloy (gamma-sterilized), typical immersion test procedure (exposed to DMEM Glutamax+ 10% FBS cell culture medium) was performed with three different exposure times in between 3 and 30 days. The results are shown in Figure 4 with four samples investigated per time point. Evaluation of the data according to ASTM NACE/ASTM G31-12a [11], Figure 5, shows the usual phenomenon of corrosion rate CR decreasing with increasing immersion time (black line). The fact that the alloy Mg-2Ag shows a nearly linear degradation behavior is indicated by the blue line in Figure 4 and Figure 5. However, this fact is not revealed by CR-analysis (slope values of blue triangles in Figure 4), which predicts the decrease in corrosion rate. A linear regression fit results in the degradation rate of 0.1 mm/a during the time interval of 3–30 days.

The phenomenon of a decrease in corrosion rate with increasing immersion times had also been reported in other studies following the standard ASTM NACE/ASTM G31-12a [15,19]. Ning et al. [19] demonstrated a decreasing corrosion rate with increasing immersion times from one hour to seven days using different cell culture media with varying cation ion concentration. Here, the drop in corrosion rate was attributed to the change in pH by the influence of cations in the medium. Precipitation of insoluble corrosion products on the surface of Mg alloys is widely regarded as the reason for decrease in corrosion rates with time [20]. However, until this day, the ambiguity exists concerning the correlation between the in vivo and in vitro corrosion rate results. The in vivo results often showed a lower corrosion compared to in vitro [8]. The approach presented in Figure 3 for determining the degradation rate is expected to provide a better in vivo—in vitro correlation provided that the immersion test is performed for duration comparable to the alloy’s intended stay as an implant. 

**Figure 6:**
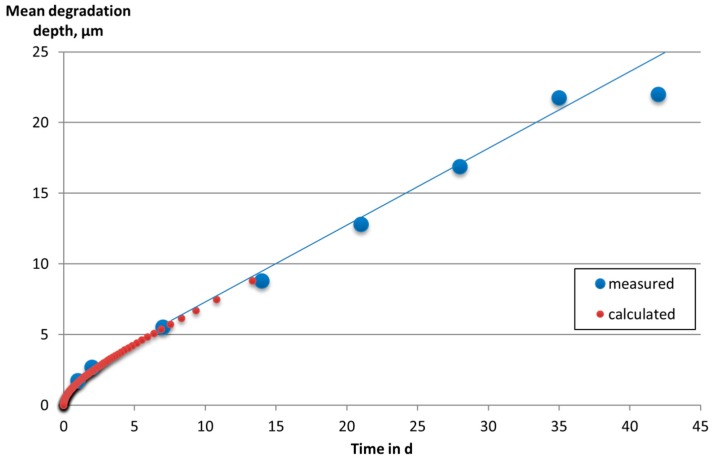
Mean degradation depth vs. time for Mg-0.3Ca, powder pressed and sintered at 642 °C, exposed to DMEM Glutamax+ 10% FBS cell culture medium (**red** dots: calculated data according to Equation 5; **blue** line: linear approximation of measured data at higher times).

In order to determine the validity of linear regression, experiments with more discrete time points are necessary. Therefore, the Mg-0.3Ca alloy was exposed to cell culture medium from one to 42 days. The resulting data is shown in Figure 6. All the data ranging from two to 42 days were fitted by linear regression at first (blue line in Figure 6), resulting in a linear degradation rate of DR = 0.51 µm/d with an intercept h_0_ = 2.2 µm. The parameter h_0_ represents the degradation depth attributing to initial reactions. The parameter ḣ_∞_ has been incorporated which attributes to degradation rate of the alloy at higher immersion times. These parameters are then inserted into an empirical two-parameter equation (Equation (5)), representing high degradation rates during early times of immersion with an exponential decay and a linearly degrading behavior as t → ∞ (higher immersion times) with a degradation rate of ḣ_∞_ as the value found by linear regression:

(5) $$h=h_{0}\cdot \left(1-e^{-\frac{h}{h_{0}}}\right)+{\dot{h}}_{\infty }\cdot t$$

The red dots in Figure 6 represent the empirical Equation (5). The data points are obtained by inverting Equation (5), i.e., for a given value of *h*, the corresponding time *t* is calculated. It can be seen that the red dots reasonably fit the measured data. The last red dot represents a point where the actual degradation rate is only 1% higher than ḣ_∞_. Beyond that, the blue line represents Equation (2) with an error less than 1%.

In Figure 6, the last measured data point is slightly higher than the previous one. It might be argued that the degradation slows down considerably after 40 days of immersion. Nevertheless, it must be pointed out that the last two measured data points are still within the scatter bar. In order to determine if the degradation actually slows down, further experiments are necessary.

Differentiation of Equation (5) with respect to time leads to an analytical expression of the implicitly time-dependent degradation rate. For differentiation, Equation (5) must be transposed to the form *t = f(h)*. Then, the derivative *dt/dh* is to be calculated. Equation (6) is obtained by *dh/dt = 1/(dt/dh)*:

(6) $$DR=\frac{dh}{dt}=\frac{{\dot{h}}_{\infty }}{1-e^{-\frac{h}{h_{0}}}}$$

**Figure 7:**
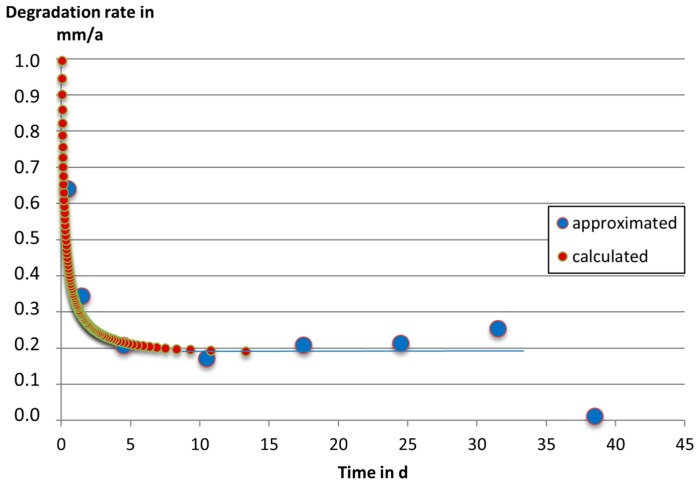
Degradation rate vs. time for Mg-0.3Ca, powder pressed and sintered at 642 °C, exposed to DMEM Glutamax+ 10% FBS cell culture medium (**red** dots: calculated data according to Equation (5) and Equation (6); **blue** dots: approximated data by step-wise numerical differentiation of measured data, Equation (4); **blue** line: nearly constant degradation rate at higher immersion times).

As shown in Equation (6), the degradation rate approaches ḣ_∞_ for t → ∞, and it tends to infinity for t → 0. Application of Equation (6) to the measured data is shown in Figure 7. Also here, for a number of discrete values of *h*, the corresponding times *t* and degradation rates *DR* are calculated. It can be seen that after about five days, the degradation rate of Mg-0.3 Ca approaches a nearly constant value of 0.51 µm/d. Before an exposure-time of 24 h, degradation is much quicker. However, the mass-change experiments are not suitable in this time frame due to the shorter immersion time compared to the drying-time afterwards. It is possible that degradation during drying could be higher than the degradation during previous incubation stage.

## 4. Conclusions

The presented approach to determine magnesium degradation under physiological conditions has several advantages:

1. Higher probability in determining the realistic corrosion rate of the alloy.
2. The amount of samples needed for this model is highly reduced compared to statistical approaches (at least *n* = 6 per time point).
3. With increasing incubation time, the risk of medium contamination gets higher. If the contamination occurs for a certain time point during immersion test, the measurement can be easily repeated from the start until the required time point without disturbing the existing setup.
4. The reduction of the amount of samples makes it possible to experiment with more alloys at the same time.

Therefore, the authors propose to include this methodology for determining the degradation rates of Mg alloys in the ongoing activities for standardization of in vitro tests.

## Figures and Tables

**Table 1:** Shape and supplier of the raw materials.

Material	Particle Size/Type	Manufacturer
Pure Mg powder	Spherical powder <45 µm	Société pour la Fabrication du Magnésium, Martigny, Switzerland
Master alloy powder (MAP) Mg-10Ca	Spherical powder 45–63 µm	Zentrum für Funktionswerkstoffe gemeinnützige GmbH, Clausthal, Germany and HZG
Pure Mg	Ingot	Xinxiang Jiuli Magnesium Co., Ltd, Xinxiang, China
Pure silver	granules	ESG Edelmetall-Handel GmbH. & Co. KG, Rheinstetten, Germany
